# Randomized Control Trial of Postnatal rhIGF-1/rhIGFBP-3 Replacement in Preterm Infants: *Post-hoc* Analysis of Its Effect on Brain Injury

**DOI:** 10.3389/fped.2020.517207

**Published:** 2020-10-09

**Authors:** Sandra Horsch, Alessandro Parodi, Boubou Hallberg, Mariya Malova, Isabella M. Björkman-Burtscher, Ingrid Hansen-Pupp, Neil Marlow, Kathryn Beardsall, David Dunger, Mirjam van Weissenbruch, Lois E. H. Smith, Mohamed Hamdani, Alexandra Mangili, Norman Barton, Luca A. Ramenghi, Ann Hellström, David Ley, ROPP-2008-01 Study Team

**Affiliations:** ^1^HELIOS Klinikum Berlin-Buch, Berlin, Germany; ^2^Department of Clinical Science, Intervention and Technology (CLINTEC), Karolinska Institutet, Stockholm, Sweden; ^3^Neonatal Intensive Care Unit, Department Mother and Child, IRCCS (Istituto di Ricovero e Cura a Carattere Scientifico) Istituto Giannina Gaslini, Genoa, Italy; ^4^Department of Clinical Sciences Lund, Radiology, Skåne University Hospital, Lund University, Lund, Sweden; ^5^Clinical Sciences, Radiology, Sahlgrenska Academy, Gothenburg University, Gothenburg, Sweden; ^6^Department of Clinical Sciences Lund, Pediatrics, Skåne University Hospital, Lund University, Lund, Sweden; ^7^Department of Academic Neonatology, UCL Elizabeth Garrett Anderson Institute for Women's Health, University College London, London, United Kingdom; ^8^Department of Paediatrics, University of Cambridge, Cambridge, United Kingdom; ^9^Department of Pediatrics, Division of Neonatology, Vrije Universiteit University Medical Center, Amsterdam UMC, Amsterdam, Netherlands; ^10^Harvard Medical School, Boston Children's Hospital, Boston, MA, United States; ^11^Global Clinical Development, Rare Metabolic Diseases, Shire, a Takeda Company, Lexington, MA, United States; ^12^Global Clinical Development, Rare Metabolic Diseases, Shire, a Takeda Company, Zurich, Switzerland; ^13^Department of Neurosciences, Rehabilitation, Ophthalmology, Genetics, Maternal and Child Health (DINOGMI), University of Genoa, Genoa, Italy; ^14^Institute of Neuroscience and Physiology, Sahlgrenska Academy, Gothenburg, Sweden

**Keywords:** neonate, brain injury, cerebral hemorrhage, recombinant human IGF-1, extremely preterm

## Abstract

**Background:** Postnatal insulin-like growth factor-1 (IGF-1) replacement with recombinant human (rh)IGF-1 and IGF binding protein-3 (rhIGF-1/rhIGFBP-3) is being studied as a potential treatment to reduce comorbidities of prematurity. We have recently reported on a phase II, multicenter, randomized, controlled trial comparing postnatal rhIGF-1/rhIGFBP-3 replacement with standard of care (SOC) in extremely preterm infants (NCT01096784). Maximum severity of retinopathy of prematurity was the primary endpoint of the trial and presence of GMH-IVH/PHI one of the pre-specified secondary endpoints. Infants therefore received serial cranial ultrasound scans (CUS) between birth and term age. In this *post-hoc* analysis we present a detailed analysis of the CUS data of this trial and evaluate the effect of postnatal rhIGF-1/rhIGFBP-3 replacement on the incidence of different kinds of brain injury in extremely preterm infants.

**Methods:** This report is an exploratory *post-hoc* analysis of a phase II trial in which infants <28 weeks gestational age were randomly allocated to rhIGF-1/rhIGFBP-3 or SOC. Serial cranial ultrasounds were performed between birth and term-equivalent age. Presence of germinal matrix hemorrhage and intraventricular hemorrhage (GMH-IVH), periventricular hemorrhagic infarction (PHI), post-hemorrhagic ventricular dilatation, and white matter injury (WMI) were scored by two independent masked readers.

**Results:** The analysis included 117 infants; 58 received rhIGF-1/rhIGFBP-3 and 59 received SOC. A trend toward less grade II–III GMH-IVH and PHI was observed in treated infants vs. SOC. A subanalysis of infants without evidence of GMH-IVH at study entry (*n* = 104) showed reduced progression to GMH-IVH in treated infants (25.0% [13/52] vs. 40.4% [21/52]; not significant). No effects of rhIGF-1/rhIGFBP-3 on WMI were observed.

**Conclusion:** The potential protective effect of rhIGF-1/rhIGFBP-3 on the occurrence of GMH-IVH/PHI appeared most pronounced in infants with no evidence of GMH-IVH at treatment start.

## Introduction

Despite advances in neonatal care and the widespread use of antenatal steroids, prematurity-related brain injuries such as germinal matrix hemorrhage and intraventricular hemorrhage (GMH-IVH), periventricular hemorrhagic infarction (PHI), post-hemorrhagic ventricular dilatation (PHVD), and white matter injury (WMI) remain common in extremely preterm infants (1–3). These events are highly related to short- and long-term adverse neurodevelopmental outcomes (4–6). It is therefore important to evaluate novel strategies to prevent brain injury in these vulnerable infants.

Postnatal levels of insulin-like growth factor-1 (IGF-1) in extremely premature infants are lower than intrauterine levels at a corresponding gestational age (GA) (7). IGF-1 is a major fetal growth factor involved in a number of processes that include metabolism, growth, and differentiation (8). Postnatal IGF-1 replacement with a complex of recombinant human (rh)IGF-1 and IGF binding protein-3 (rhIGF-1/rhIGFBP-3) is being studied as a potential treatment to reduce comorbidities associated with premature birth. A phase II, multicenter, randomized, controlled trial recently compared postnatal rhIGF-1/rhIGFBP-3 replacement with standard of care (SOC) in extremely preterm infants (NCT01096784) (9). The maximum severity of retinopathy of prematurity was the primary endpoint of the trial and presence of GMH-IVH detected by cranial ultrasound scans (CUS) was a pre-specified secondary endpoint. Results of that trial showed a trend toward reduction in IVH favoring active treatment. The study was not powered for a reduction in IVH and some study infants already had evidence of GMH-IVH at study entry, which also may have influenced the results. The reason that some study infants had evidence of GMH-IVH at study entry is because the baseline scan was read locally to allow enrollment decisions within the first 24 h after birth. The effect of postnatal IGF-1 replacement on incidence of PHVD and WMI detected by CUS has not yet been reported.

We conducted *post-hoc* analyses of data from that trial to clarify the findings relative to GMH-IVH, and to further evaluate the effect of postnatal rhIGF-1/rhIGFBP-3 replacement on the incidence of prematurity-related brain injury (GMH-IVH, PHI, PHVD, and WMI), as assessed by CUS.

## Methods

### Study Design and Patient Population

The methods, study design, and results from the primary analyses of the phase II study were reported previously (9). In brief, this was a multicenter, randomized, SOC concurrent control, assessor-masked study of rhIGF-1/rhIGFBP-3 (mecasermin rinfabate, 50 μg/ml solution) in extremely preterm infants (ClinicalTrials.gov, NCT01096784). Eligible infants had a GA at birth ranging from 23 weeks + 0 days to 27 weeks + 6 days. Exclusion criteria included detectable gross malformation, known or suspected chromosomal abnormality, clinically significant neurological disease, GMH-IVH grade II or III, or PHI (infants with grade I GMH-IVH were included). Infants in the active treatment group received a standardized dosage of 250 μg/kg per day of rhIGF-1/rhIGFBP-3 via continuous intravenous infusion in addition to SOC from ≤24 h of birth until a post-menstrual age (PMA) of 29 weeks + 6 days. Infants in the control group received SOC based on their individual medical needs and according to local protocols. The primary endpoint of the phase II study was maximum severity of retinopathy of prematurity (ROP). Secondary endpoints included IVH, time to discharge from neonatal care, bronchopulmonary dysplasia, and growth parameters (9). A *post-hoc* analysis was conducted to further explore the phase II study findings relative to IVH.

Written informed consent was provided by all infants' parents/guardians. The study was reviewed/approved by all relevant institutional review boards/independent ethics committees of all participating centers.

### Detection and Assessment of Brain Injury on CUS

As part of the phase II trial, CUS examinations were performed to detect and assess cerebral hemorrhage at study entry (day 0); at postnatal days 3, 7, 14, and 21 (±1 day); and at PMA of 40 weeks (± 4 days). As initially reported, a single reader (masked to treatment) evaluated all ultrasound images for the highest grade of GMH-IVH, according to Papile and Bowerman methods (10, 11). No IVH and grade I IVH were grouped together. As a *post-hoc* follow up, CUS was re-examined for GMH-IVH and PHI for each scan by two independent readers, who were masked to treatment allocation. In addition, CUS images were analyzed for the presence of PHVD and WMI. GMH-IVH was graded according to Volpe (12); PHI was graded by localization and extent of the lesion according to Dudink (13). PHVD was measured by anterior horn width (AHW) according to Davies, and graded as follows: normal, <3 mm; mild, AHW 3 to <5 mm; moderate, AHW 5–10 mm; severe, AHW >10 mm [(14); Table 1]. WMI was schematically described according to Govaert and de Vries (15), with presentation as persistent periventricular hyperechogenicity, white matter loss, or cystic periventricular leukomalacia (PVL). White matter loss was identified as ventricular dilatation without hemorrhage on CUS. WMI was scored using a four-grade classification. A seven-grade brain injury severity score was developed by one of the authors (SH) for use in the current study (not previously published). The brain injury severity score utilized a classification system where each grade reflected a greater degree of brain injury (Table 1). The brain injury severity score was recorded for each infant at 40 weeks PMA. Information on surgical intervention in infants with PHVD (e.g., shunt, Rickham device) was collected prospectively. Any discrepancy between the two readers was resolved by consensus agreement.

**Table 1:** Cranial ultrasound image grading systems.

**GMH-IVH graded according to Volpe method** **(****12****)**	
**Severity**	**Description**
Grade I	GMH with no or minimal IVH (<10% of ventricular area on parasagittal view)
Grade II	IVH in 10–50% of ventricular area on parasagittal view
Grade III	IVH in >50% of ventricular area on parasagittal view; usually distends lateral ventricle
Grade IV (IVH and PHI)	IVH compounded by hemorrhagic venous infarction in the periventricular white matter
**PHI graded according to Dudink method** **(****13****)**	
**Localization**	Caudate vein infarct
	Temporal vein infarct
	Anterior terminal vein infarct
	Complete terminal vein infarct
**Severity**	**Description**
Limited PHI	Only caudate vein or temporal vein affected; small anterior terminal vein infarction
Extensive PHI	Complete terminal vein infarction or combination of caudate vein, temporal vein, anterior vein infarctions
**PHVD graded according to Davies method** **(****14****)**	
**Severity**	**Description**
0	Normal, AHW <3 mm
1	Mild, AHW 3 to <5 mm
2	Moderate AHW 5–10 mm
3	Severe AHW >10 mm
**White matter injury**	
**Schematic description**	
•	Persistent periventricular hyperechogenicity, no cysts, no obvious white matter loss (white matter loss was identified as ventricular dilatation without hemorrhage)
•	Persistent periventricular hyperechogenicity evolving into diffuse white matter loss, but no cysts
•	Persistent periventricular hyperechogenicity evolving into small localized frontoparietal cystic lesions (limited cystic PVL)
•	Persistent periventricular hyperechogenicity evolving into extensive cystic lesions (extensive cystic PVL)
**Scoring**^a^	**Description**
0	Persistent periventricular hyperechogenicity or punctate lesions without overt white matter loss, no cysts
1	Persistent periventricular hyperechogenicity or extensive punctate lesions evolving in a white matter loss without cysts
2	Limited cystic PVL
3	Extensive cystic PVL
**Brain injury severity score**	
0	No brain abnormalities
1	GMH, periventricular hyperechogenicity without white or gray matter loss, and mild-moderate cerebral injury
2	IVH II, mild PHVD, stroke of a perforating artery
3	IVH III, persistent moderate PHVD without shunt or Rickham device, persistent periventricular hyperechogenicities with diffuse white and/or gray matter loss at term cranial ultrasound
4	Limited PHI, limited PVL, PHVD with shunt or Rickham device, anterior cerebral artery stroke, posterior cerebral artery stroke, severe cerebellar injury
5	Unilateral extensive PHI, extensive cystic PVL, mild cerebral artery stroke, severe global brain atrophy^b^
6	Bilateral extensive PHI

**Figure 1:**
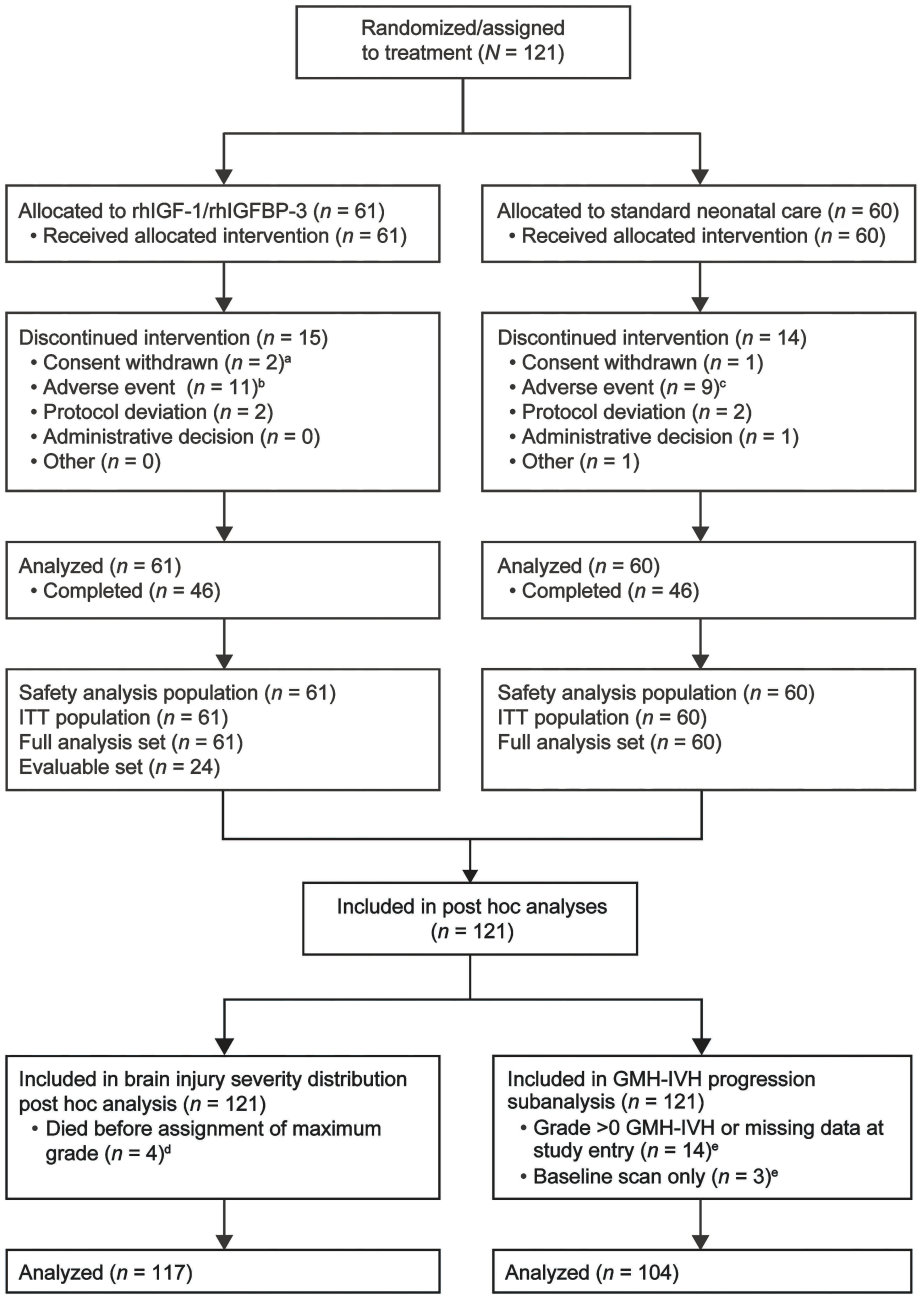
Flowchart of infants included in the phase II study (9), the *post-hoc* analysis, and the subanalysis. GMH-IVH, germinal matrix hemorrhage and intraventricular hemorrhage; rhIGF-1, recombinant human insulin-like growth factor-1; rhIGFBP-3, recombinant human insulin-like growth factor binding protein-3. ^a^One infant had a serious adverse event with fatal outcome, but the primary reason for discontinuation was withdrawal of consent. ^b^All infants discontinued due to a serious adverse event with fatal outcome. ^c^Seven of nine discontinuations were due to serious adverse events with fatal outcome. ^d^For the distribution analysis (*n* = 117), each infant was classified based on the maximum grade of GMH-IVH observed between day 0 and week 40 during a joint masked consensus review of all available scans for that infant; one infant receiving standard of care and three infants receiving rhIGF-1/rhIGFBP-3 died within 72 h of randomization and did not have an assigned maximum grade. ^e^The progression analysis (*n* = 104) was based on a comparison of longitudinal scans with the baseline grade 0 scan for each eligible infant; 17 infants were excluded for the progression analysis: grade 0 → missing (*n* = 3); grade I → grade I (*n* = 4); grade I → grade II (*n* = 2); grade II → grade II (*n* = 3); grade II → grade IV (*n* = 1); grade III → grade IV (*n* = 1); grade IV → grade IV (*n* = 1); missing → grade 0 (*n* = 1); missing → grade III (*n* = 1). Reprinted from Ley et al. (9), Copyright 2019, with permission from Elsevier. https://www.sciencedirect.com/science/article/pii/S0022347618315403.

*AHW, anterior horn width; GMH, germinal matrix hemorrhage; IVH, intraventricular hemorrhage; PHI, periventricular hemorrhagic infarction; PHVD, post-hemorrhagic ventricular dilatation; PVL, periventricular leukomalacia*.

a *Grade 0 and grade I persistent periventricular hyperechogenicity were combined for presentation in Figure 1; grade II and grade III were combined for cystic PVL*.

b *Severe global brain atrophy defined as a combination of global loss of gray and white matter, delayed cortical folding, and enlarged lateral ventricles and subarachnoid spaces*.

### Statistical Analysis

The current study included *post-hoc* analysis of brain injury severity distribution, and a further subanalysis on GMH-IVH progression in those infants without hemorrhage on the baseline CUS. The severity distribution for GMH-IVH analysis included all eligible infants in the phase II study. The distribution of GMH-IVH according to treatment group was determined from the maximum-grade hemorrhage observed for each infant in the study population after randomization. A consensus maximum GMH-IVH grade for each infant was defined based on the highest grade of GMH-IVH observed by joint masked reader review of all scans performed for that infant between day 0 and PMA 40 weeks (even in the event that some scans were missing). In the subanalysis, the preventative effect of treatment with rhIGF-1/rhIGFBP-3 on GMH-IVH was assessed based on the progression of cerebral hemorrhage during the study among infants with no evidence of GMH-IVH or PHI (classified as grade 0 GMH-IVH) at study entry. Progression to GMH-IVH in infants with no evidence of hemorrhage at baseline was analyzed based on the highest grade identified at any subsequent scan after the baseline scan. The final analysis in the current study included two different subgroups; 117 infants were included in the *post-hoc* analysis for brain injury, and 104 infants were included in the GMH-IVH progression analysis.

The Fisher exact test was performed to test the significance of the difference between the rhIGF-1/rhIGFBP-3 treatment and SOC groups. A *p* ≤ 0.05 was regarded as significant. The grade of GMH-IVH (grades I–III) or PHI was summarized descriptively by treatment group and GA strata. Weighted kappa statistics were used to measure interrater agreement between the two readers. No power calculations were performed for comparing GMH-IVH in this *post-hoc* analysis, since it was a secondary endpoint; the trial was only powered for the primary endpoint of maximum severity of retinopathy of prematurity in the primary phase II study.

## Results

### Brain Injury Severity Distribution Analysis (*n* = 117)

A total of 121 infants were enrolled in the original phase II trial, and details of patient disposition among these infants have been previously reported (9). In the current study, 117 infants were assigned a maximum grade of GMH-IVH and were included in the analysis for presence of brain injury (Figure 1). Four infants died within 72 h of randomization and were not assigned a maximum grade. The cause of death was respiratory failure in each circumstance; baseline CUS revealed no evidence of hemorrhage. Fifty-eight of 117 infants received rhIGF-1/rhIGFBP-3 and 59 received SOC. Thirty-two of 58 (55.2%) treated infants and 31 of 59 (52.5%) control infants were born before 26 weeks GA (Table 2).

**Table 2:** Demographic characteristics and maternal/perinatal histories of infants included in the *post-hoc* analysis (*n* = 117).

**Characteristic**	**Standard of care**	**rhIGF-1/rhIGFBP-3**
	**(*n* = 59)**	**(*n* = 58)**
**Sex**, ***n*** **(%)**		
Male	38 (64.4)	38 (65.5)
Female	21 (35.6)	20 (34.5)
**GA group**, ***n*** **(%)**		
<26 weeks	31 (52.5)	32 (55.2)
≥26 weeks	28 (47.5)	26 (44.8)
**GA (weeks)**		
Mean (SD)	25 (1)	26 (1)
**Weight at birth (g)**		
Mean (SD)	803 (175)	782 (186)
**Race**, ***n*** **(%)**		
Asian	5 (8.5)	4 (6.9)
Black or African American	9 (15.3)	4 (6.9)
White	41 (69.5)	47 (81.0)
Other	4 (6.8)	3 (5.2)
**Mode of delivery**, ***n*** **(%)**		
Vaginal	27 (45.8)	24 (41.4)
Cesarean section	32 (54.2)	34 (58.6)
Maternal infections, *n* (%)	14 (23.7)	10 (17.2)
Clinical chorioamnionitis, *n* (%)	6 (10.2)	9 (15.5)
Maternal antibiotics, *n* (%)	38 (64.4)	29 (50.0)
Antenatal steroids, *n* (%)	59 (100)	58 (100.0)
Fertility therapy, *n* (%)	8 (13.6)	8 (13.8)
*In vitro* fertilization	6 (10.2)	8 (13.8)
Ovulation stimulation	2 (3.4)	0
Preterm labor, *n* (%)	52 (88.1)	47 (81.0)
Preterm premature rupture of	20 (33.9)	16 (27.6)
membranes, *n* (%)		
Preeclampsia, *n* (%)	5 (8.5)	7 (12.1)
Apgar score at 5 min		
Median (range)	7.0 (2.0–10.0)	8.0 (1.0–10.0)

*GA, gestational age; rhIGF-1, recombinant human insulin-like growth factor-1; rhIGFBP-3, recombinant human insulin-like growth factor binding protein-3; SD, standard deviation*.

### GMH-IVH or PHI

**Figure 2:**
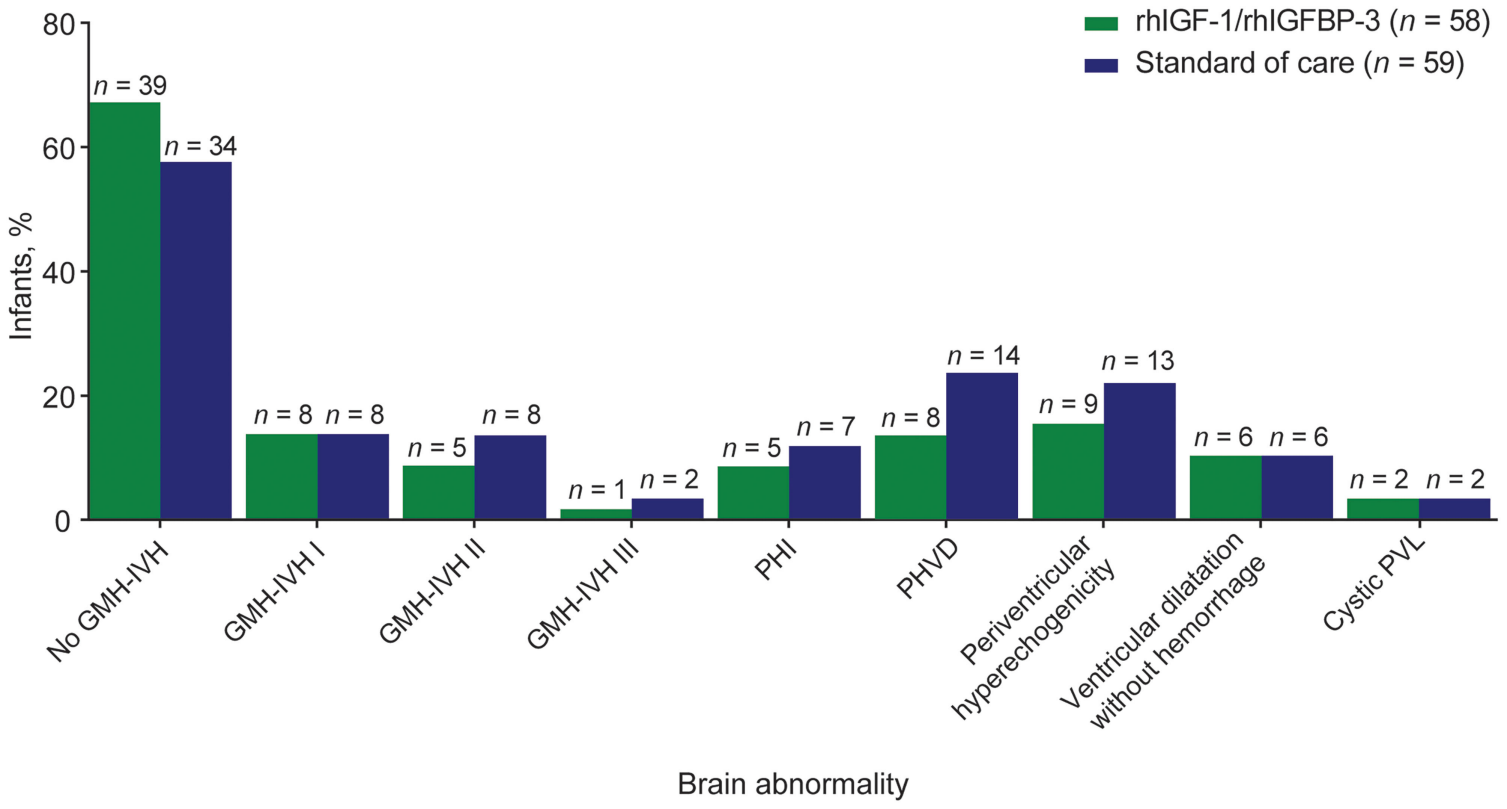
Distribution of brain abnormalities among extremely preterm infants receiving rhIGF-1/rhIGFBP-3 or standard of care.^a^ GMH-IVH, germinal matrix hemorrhage and intraventricular hemorrhage; PHI, periventricular hemorrhagic infarction; PHVD, post-hemorrhagic ventricular dilatation; PVL, periventricular leukomalacia; rhIGF-1, recombinant human insulin-like growth factor-1; rhIGFBP-3, recombinant human insulin-like growth factor binding protein-3. ^a^Based on the maximum-grade hemorrhage for each infant observed in cranial ultrasound on study days 0, 3, 7, 14, and 21, and at 40 weeks post-menstrual age.

**Figure 3:**
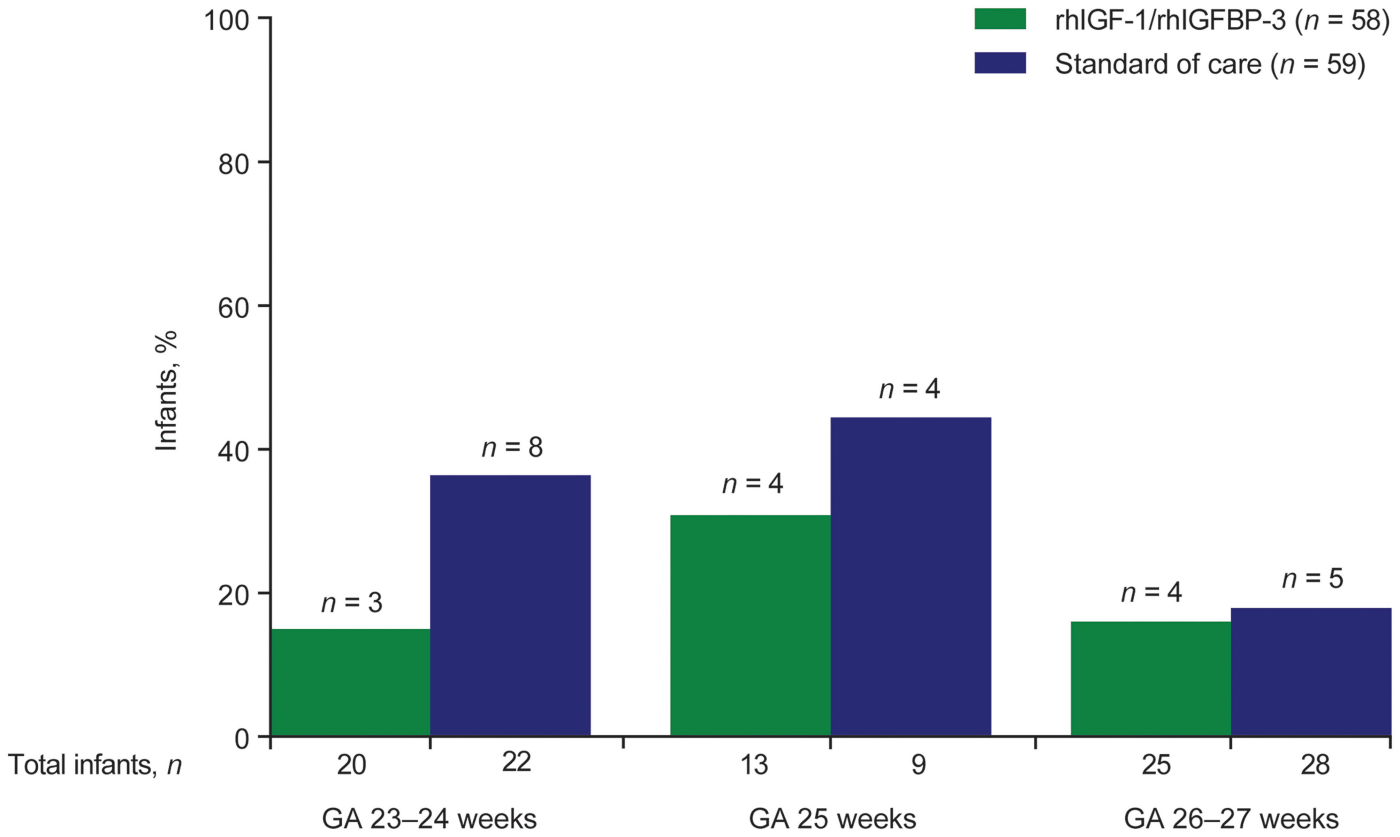
Distribution of GMH-IVH grade II–III or PHI among infants assigned a maximum grade (*n* = 117), by treatment group and gestational age. GA, gestational age; GMH-IVH, germinal matrix hemorrhage and intraventricular hemorrhage; PHI, periventricular hemorrhagic infarction; rhIGF-1, recombinant human insulin-like growth factor-1; rhIGFBP-3, recombinant human insulin-like growth factor binding protein-3.

A smaller proportion of treated infants had grade II–III GMH-IVH or PHI, compared with control infants; the differences were not statistically significant (Figure 2). Across all GAs, a higher frequency of grade II–III GMH-IVH or PHI was observed among infants receiving SOC vs. rhIGF-1/rhIGFBP-3 (Figure 3). Among infants <25 weeks GA, a smaller proportion of rhIGF-1/rhIGFBP-3–treated infants had grade II–III GMH-IVH or PHI compared with controls (15.0% [*n* = 3/20] vs. 36.4% [*n* = 8/22], respectively; not statistically significant).

### PHVD and WMI

Overall, the proportion of infants with PHVD was lower among treated infants vs. controls (not statistically significant; Figure 2). Mild PHVD (AHW 3–5 mm) occurred in 1.7% (*n* = 1/58) of infants in the treated group and 15.3% (*n* = 9/59) in the SOC group. Moderate PHVD (AHW 5–10 mm) occurred in 8.6% (*n* = 5/58) in the treated group and 6.8% (*n* = 4/59) in the SOC group. Severe PHVD (AHW >10 mm) was low in both the treated and SOC groups: 3.4% (*n* = 2/58) vs. 1.7% (*n* = 1/59), respectively (these findings were not statistically significant). A smaller proportion of infants in the rhIGF-1/rhIGFBP-3 group had periventricular hyperechogenicity than among controls: 15.5% (*n* = 9/58) vs. 22% (*n* = 13/59), respectively (not statistically significant; Figure 2). The proportion of treated vs. control infants with ventricular dilatation without hemorrhage (mild and moderate) was equal (10.3% [*n* = 6/58] vs. 10.2% [*n* = 6/59], respectively). No infants had severe dilatation without hemorrhage. The proportion of treated vs. control infants with cystic PVL (limited and extensive) was equal (3.4% [*n* = 2/58] vs. 3.4% [*n* = 2/59], respectively).

### Brain Injury Score

**Figure 4:**
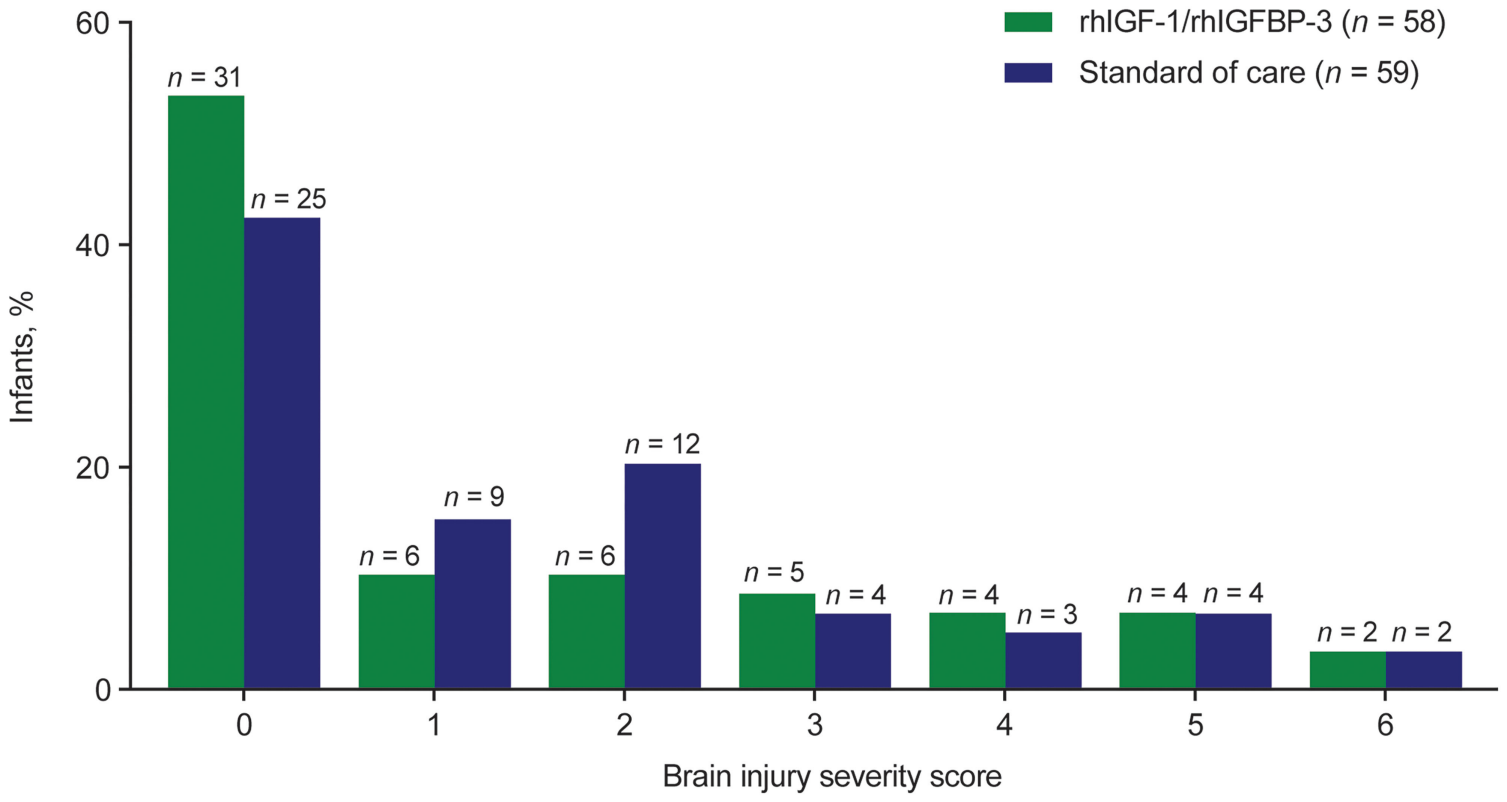
Brain injury severity score among infants assigned a maximum grade (*n* = 117), by treatment group. rhIGF-1 recombinant human insulin-like growth factor-1, rhIGFBP-3 recombinant human insulin-like growth factor binding protein-3.

Compared with control infants, a higher proportion of infants in the rhIGF-1/rhIGFBP-3 group had a brain injury severity score of 0 (53.4 % [*n* = 31/58] vs. 42.4% [*n* = 25/59], respectively; Figure 4). The proportion of infants with a severity score ≥3 was slightly higher among treated infants (25.9% [*n* = 15/58]) than control infants (22.0% [*n* = 13/59]). These differences were not statistically significant.

Overall, findings from the brain injury severity distribution analysis among 117 infants showed no significant effect on brain injury among treated infants vs. infants receiving SOC.

### Subanalysis on the Preventative Effect of rhIGF-1/rhIGFBP-3 on GMH-IVH (*n* = 104)

Of the total phase II study population (*n* = 121), 107 infants had no evidence of GMH-IVH on CUS at study entry (14 infants had evidence of GMH-IVH grade >0 or missing data at study entry). Three infants had a baseline scan only and were excluded from the subanalysis. One infant, excluded from the distribution analysis due to lack of assignment of maximum grade owing to poor quality of day 3 CUS, was included in the progression analysis. A total of 104 infants were, therefore, included in the subanalysis of GMH-IVH progression (Figure 1). Of these infants with no GMH-IVH at study entry, 70 remained hemorrhage free over the course of the study (39 infants in the rhIGF-1/rhIGFBP-3 group; 31 in the SOC group), while 34 developed GMH-IVH (13 in the rhIGF-1/rhIGFBP-3 group; 21 in the SOC group; Table 3).

**Table 3:** Demographics, characteristics, and maternal/perinatal histories of infants with no GMH-IVH at study entry who either remained hemorrhage free or developed GMH-IVH after study entry (*n* = 104).

**Characteristic**	**Infants who remained GMH-IVH free (*****n*** **=** **70)**		**Infants who developed GMH-IVH (*****n*** **=** **34)**	
	**Standard of care** ** (*n* = 31)**	**rhIGF-1/rhIGFBP-3** ** (*n* = 39)**	**Standard of care** ** (*n* = 21)**	**rhIGF-1/rhIGFBP-3** ** (*n* = 13)**
**Sex**, ***n*** **(%)**				
Male	18 (58.1)	26 (66.7)	16 (76.2)	8 (61.5)
Female	13 (41.9)	13 (33.3)	5 (23.8)	5 (38.5)
**GA group**, ***n*** **(%)**				
<26 weeks	11 (35.5)	19 (48.7)	16 (76.2)	9 (69.2)
≥26 weeks	20 (64.5)	20 (51.3)	5 (23.8)	4 (30.8)
**GA (weeks)**				
Mean (SD)	26 (1)	26 (1)	25 (1)	25 (1)
**Weight at birth (g)**				
Mean (SD)	836 (182)	779 (173)	747 (145)	816 (221)
**Race**, ***n*** **(%)**				
Asian	2 (6.5)	3 (7.7)	3 (14.3)	1 (7.7)
Black or African American	5 (16.1)	1 (2.6)	4 (19.0)	3 (23.1)
White	22 (71.0)	32 (82.1)	13 (61.9)	9 (69.2)
Other	2 (6.5)	3 (7.7)	1 (4.8)	0
**Mode of delivery**, ***n*** **(%)**				
Vaginal	14 (45.2)	14 (35.9)	8 (38.1)	8 (61.5)
Cesarean section	17 (54.8)	25 (64.1)	13 (61.9)	5 (38.5)
Maternal infections, *n* (%)	4 (12.9)	5 (12.8)	8 (38.1)	4 (30.8)
Clinical chorioamnionitis, *n* (%)	2 (6.5)	3 (7.7)	2 (9.5)	4 (30.8)
Maternal antibiotics, *n* (%)	17 (54.8)	18 (46.2)	17 (81.0)	8 (61.5)
Antenatal steroids, *n* (%)	31 (100)	39 (100)	21 (100)	13 (100)
Fertility therapy, *n* (%)	7 (22.6)	4 (10.3)	1 (4.8)	3 (23.1)
*In vitro* fertilization	5 (16.1)	4 (10.3)	1 (4.8)	3 (23.1)
Ovulation stimulation	2 (6.5)	0	0	0
Preterm labor, *n* (%)	27 (87.1)	29 (74.4)	19 (90.5)	13 (100)
Premature rupture of membranes, *n* (%)	9 (29.0)	9 (23.1)	8 (38.1)	6 (46.2)
Preeclampsia, *n* (%)	2 (6.5)	6 (15.4)	2 (9.5)	0
Apgar score at 5 min				
Median (range)	7.0 (2.0–10.0)	8.0 (3.0–10.0)	7.0 (4.0–10.0)	6.0 (1.0–9.0)

*GA, gestational age; GMH-IVH, germinal matrix hemorrhage and intraventricular hemorrhage; rhIGF-1, recombinant human insulin-like growth factor-1; rhIGFBP-3, recombinant human insulin-like growth factor binding protein-3; SD, standard deviation*.

Among infants in the SOC group whose mothers had infections, a significantly higher proportion developed GMH-IVH than remained GMH-IVH free (38.1% [*n* = 8/21] vs. 12.9% [*n* = 4/31], respectively; *p* = 0.05). The difference in the proportion of treated infants with maternal infections who developed hemorrhages or remained hemorrhage free was not statistically significant. A higher frequency of clinical chorioamnionitis and maternal antibiotic use was observed among infants who developed IVH/PHI in both the SOC and rhIGF-1/rhIGFBP-3 treatment groups, compared with infants who remained hemorrhage free (not statistically significant; Table 3).

### GMH-IVH Progression

**Figure 5:**
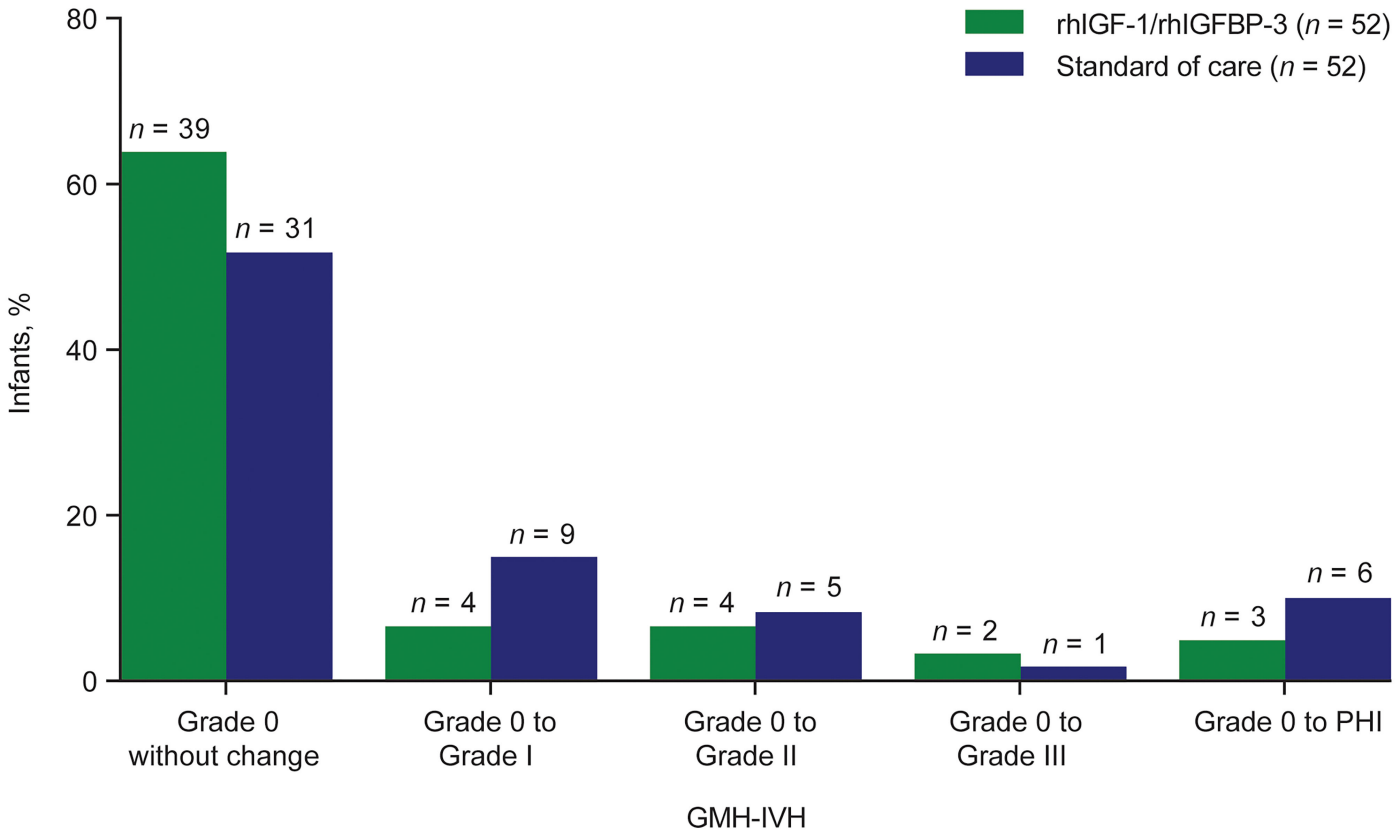
Progression of GMH-IVH among infants with no evidence of GMH-IVH on cranial ultrasound at study entry (*n* = 104).^a^ GMH-IVH, germinal matrix hemorrhage and intraventricular hemorrhage; PHI, periventricular hemorrhagic infarction; rhIGF-1, recombinant human insulin-like growth factor-1; rhIGFBP-3, recombinant human insulin-like growth factor binding protein-3. ^a^Seventeen infants were excluded for the progression analysis: grade 0 → missing (*n* = 3); grade I → grade I (*n* = 4); grade I → grade II (*n* = 2); grade II → grade II (*n* = 3); grade II → grade IV (*n* = 1); grade III → grade IV (*n* = 1); grade IV → grade IV (*n* = 1); missing → grade 0 (*n* = 1); missing → grade III (*n* = 1).

Among 104 infants who had no evidence of GMH-IVH on CUS at study entry, there was a trend toward less progression to GMH-IVH grade I–III or PHI among treated infants compared with SOC (25.0% [*n* = 13/52] vs. 40.4% [*n* = 21/52], respectively; *p* = 0.14; percentage risk difference, −15.4%; confidence interval [CI], −34.6 to 4.8%; Figure 5).

## Discussion

To our knowledge, this is the first multicenter, randomized, controlled trial to evaluate the effect of postnatal rhIGF-1/rhIGFBP-3 replacement on brain injury in extremely preterm infants, as assessed by serial CUS. Among the full population in the current study (*n* = 117), we observed a lower prevalence of grade II–III GMH-IVH and PHI in infants receiving rhIGF-1/rhIGFBP-3 vs. infants receiving SOC. The prevalence of grade I GMH-IVH and WMI (cystic PVL and white matter loss) was broadly comparable between groups.

*Post-hoc* analysis of the serial CUS imaging data from the phase II trial (9) by two independent central readers revealed that 12 infants (6 in the treatment group, 6 in the control group) had pre-existing GMH-IVH before treatment with rhIGF-1/rhIGFBP-3 commenced, which may have attenuated the observed protective effect of rhIGF-1/rhIGFBP-3 replacement on the occurrence of GMH-IVH. Therefore, we performed a further exploratory *post-hoc* analysis, including a subcohort of infants without pre-existing GMH-IVH (*n* = 104), in order to study the potentially preventive effect of rhIGF-1/rhIGFBP-3 replacement. In this subcohort, 25.0% in the treatment group vs. 40.4% in the SOC group developed GMH-IVH or PHI. Although not statistically significant, we believe that the potentially beneficial effect of rhIGF-1/rhIGFBP-3 replacement in preventing GMH-IVH is more pronounced in the *post-hoc* analysis than we were able to demonstrate in the clinical trial. No power calculations were performed for comparing GMH-IVH (the study was powered for the retinopathy of prematurity endpoint in the primary study only). Dose-response characteristics for this potentially beneficial effect will be further explored in a larger clinical trial that is currently underway (EudraCT number: 2018-001393-16). If the protective effect of rhIGF-1/rhIGFBP-3 can be confirmed in a larger cohort of preterm infants, early administration of the drug may be beneficial to reduce GMH-IVH occurrence.

While severe GMH-IVH (grade III and PHI) is commonly used as an outcome parameter in clinical trials, low-grade GMH-IVH (i.e., grade I and II) is not always considered a relevant neonatal morbidity. Indeed, it has become customary to inform parents that an uncomplicated, limited GMH-IVH has no relevance in relation to long-term outcomes. However, recent data have associated low-grade hemorrhage with neurodevelopmental impairment in preterm infants (16). A meta-analysis by Mukerji et al. found an increased risk for moderate to severe neurodevelopmental impairment at 18–24 months (adjusted odds ratio, 1.39; 95% CI, 1.09–1.77) in infants with mild (grade I and/or II) periventricular/intraventricular hemorrhage compared with infants without hemorrhage (17). Even though the meta-analysis was based on a limited amount of data, low-grade GMH-IVH might in the future need to be taken into account when considering clinical outcome.

The exact neurobiological basis of the adverse effect of uncomplicated low-grade GMH-IVH on neurodevelopmental outcome still needs to be elucidated, but it is likely multifactorial, and there are several pathogenetic mechanisms that have been related to the observed brain injury after low-grade GMH-IVH. The destruction of the germinal matrix itself may result in a relevant loss of glial precursor cells, leading to impaired myelination, and cortical development (18, 19). Low-grade GMH-IVH can be followed by abnormal microstructural alterations in periventricular and subcortical white matter (20). Even limited amounts of intraventricular blood can further trigger inflammation in adjacent white matter through activated microglia, passage of red blood cells, and red blood cell degradation; the resulting perilesional tissue injury may be secondary to free radical release and the presence of free iron (21–24). In this context, we believe it is important to include low-grade GMH-IVH as an outcome variable, although careful long-term neurodevelopmental follow up of larger cohorts is needed to prove this assumption.

Infants who experience PHVD after GMH-IVH carry a higher risk of adverse neurodevelopmental outcomes than infants without PHVD (25–27). In our cohort, 13.8% of infants receiving rhIGF-1/rhIGFBP-3 developed some degree of PHVD, compared with 23.7% of infants receiving SOC. However, the difference was mainly due to an increased incidence of mild PHVD in the control group, which is in line with the finding of a higher prevalence of low-grade GMH-IVH in the control group. The incidence of severe PHVD (AHW >10 mm) was low, and comparable in both groups (2 infants in the treatment group vs. 1 infant in the control group). Therefore, the clinical relevance of the observed difference in subtle cases of PHVD between groups remains speculative.

WMI is common in preterm infants (28, 29). The cystic form of WMI, also known as cystic PVL, is highly associated with cerebral palsy (28, 30). It can be reliably detected by serial CUS imaging (31). Today, due to advances in neonatal care, it has become a rare disease (32). The incidence of cystic PVL was 3.4% in each of the study groups (rhIGF-1/rhIGFBP-3 and SOC) in the current study, which is comparable to data from population-based cohorts (1, 3). The non-cystic form of WMI, the more common type of WMI in preterm infants today, can present on CUS as persisting periventricular hyperechogenicity. The incidence of periventricular hyperechogenicity was 15.5% in the treatment group vs. 22.0% in the SOC group in the current study. Non-cystic WMI can lead to impaired brain growth and development and brain atrophy. One of the sonographic signs of impaired brain development or loss of gray and white matter volume is ventricular dilatation without hemorrhage on the term-age ultrasound. This finding on term-age CUS has been shown to correlate to long-term outcomes (33, 34). The prevalence of ventricular dilatation without hemorrhage was identical in both groups in the current trial (rhIGF-1/rhIGFBP-3 10.3% vs. SOC 10.2%). Therefore, we could not find evidence of either adverse or beneficial effects of rhIGF-1/rhIGFBP-3 replacement on cystic or non-cystic WMI with serial CUS.

The present data suggest that postnatal replacement therapy with rhIGF-1/rhIGFBP-3 may have a beneficial effect on causal mechanisms involved in the development of preterm GMH-IVH. The rupture of vessels leading to GMH-IVH has been attributed to an increased vulnerability of the germinal matrix vasculature to fluctuations in cerebral blood flow (35). The vasculature of the germinal matrix is in a highly proliferative phase and exhibits a paucity of pericytes and an immature basal lamina low in fibronectin (35). Further, circulating IGF-1 deficiency has been shown to compromise the structural integrity of the cerebral vasculature, resulting in decreased cerebral capillary density and impaired cerebral myogenic autoregulation in preclinical studies (36–38). The relationship between decreased circulating levels of IGF-I and structural or functional aspects of the immature blood-brain barrier remain to be elucidated.

The strength of this study is the multicenter, randomized, controlled study design. Centers in five European countries and the United States participated in the trial, which underlines the generalizability of our findings. Furthermore, CUS was performed serially from birth to term age and analyzed independently by two masked readers, increasing reliability in detection of not only mild hemorrhage, but also cystic and non-cystic forms of WMI as well as severe brain atrophy.

Limitations include the relatively small sample size in our treated and SOC cohorts, which could be a possible explanation as to why a number of our results did not reach significance. The trial was powered for the primary endpoint: maximum severity of retinopathy of prematurity. Presence of GMH-IVH was one of the pre-specified secondary endpoints, but the study was not powered for IVH reduction, nor for reduction of the other types of prematurity-related brain injury. It is therefore important that a larger clinical trial is underway that is planning to enroll ~600 infants (EudraCT number: 2018-001393-16; NCT03253263). An additional consideration is that CUS was performed using the anterior fontanel as an acoustic window. Additional visualization of the posterior fossa via mastoid fontanel would have improved detection of cerebellar injury (39, 40) but was not part of the initial study protocol. Another limitation is that brain injury was evaluated by CUS only. Magnetic resonance imaging (MRI) as a complementary imaging modality would have certainly increased the sensitivity and accuracy of the detection of prematurity-related brain injury, and would have allowed detailed segmentation of different brain regions and volumetric studies, as well as quantification of white matter changes. However, acquiring high quality MRI data in a multicenter study setting can be challenging compared to sequential CUS. The advantage of CUS is that it is a bedside tool that is nearly universally available in neonatal intensive care units, and allows frequent serial imaging with minimal disturbance of the infants and thereby gives valuable information on the timing and evolution of brain injury. This can be crucial especially in intervention studies like ours where timing of brain injury in relation to drug application is an important aspect.

## Conclusions

In this first multicenter, randomized, controlled trial comparing rhIGF-1/rhIGFBP-3 replacement therapy with standard treatment in extremely preterm infants, a trend toward less grade II–III GMH-IVH and PHI in the treatment group was observed across GAs. The potential protective effect of rhIGF-1/rhIGFBP-3 was most pronounced in infants with no evidence of GMH-IVH at the start of treatment. No effects of rhIGF-1/rhIGFBP-3 replacement on WMI were observed. These results support further investigation of the potential beneficial effects of rhIGF-1/rhIGFBP-3 replacement in a larger cohort of extremely preterm infants.

## Data Availability Statement

The datasets, including the redacted study protocol, redacted statistical analysis plan, and individual participants data supporting the results reported in this article, will be available three months after the submission of a request, to researchers who provide a methodologically sound proposal. The data will be provided after its de-identification, in compliance with applicable privacy laws, data protection and requirements for consent and anonymization.

## Ethics Statement

The studies involving human participants were reviewed and approved by EC/IRB and regulatory agency (as appropriate). Written informed consent to participate in this study was provided by the participants' legal guardian/next of kin.

## Author Contributions

SH, AP, BH, MM, IB-B, IH-P, MH, AM, NB, LR, AH, and DL made substantial contributions to conception and design, acquisition of data, or analysis and interpretation of data. All authors drafted the article or revised it critically for important intellectual content, approved the final manuscript as submitted, and agree to be accountable for all aspects of the work.

## Conflict of Interest

SH, AP, MM, KB, MW, and LS have received consulting fees from Shire, a Takeda company; BH has received consulting fees from Premacure AB and Shire, a Takeda company; IH-P and DL hold stock/stock options in Premalux AB, and have received consulting fees from Shire, a Takeda company; NM has received consulting fees from Shire, a Takeda company, and partial funding from the Department of Health's National Institute for Health Research Biomedical Research Centre's funding scheme at University College London Hospitals/University College London; DD has received consulting fees from Shire, a Takeda company, and has received consulting fees from Ipsen regarding other indications for IGF-1 therapies; MH was employed by Shire, a Takeda company, at the time of the study and *post-hoc* analysis; AM and NB were employed by Shire, a Takeda company; LR has received consulting fees and research support from Shire, a Takeda company; AH holds stock/stock options in Premalux AB, and has received consulting fees from Shire, a Takeda company; IB-B declares that the research was conducted in the absence of any commercial or financial relationships that could be construed as a potential conflict of interest.
